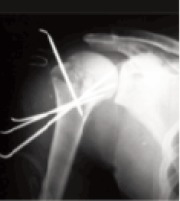# Long Term Results of PHILOS Plating and Percutaneous K-Wire Fixation in Proximal Humerus Fractures in The Elderly

**DOI:** 10.5704/MOJ.1403.010

**Published:** 2014-03

**Authors:** GS Jaura, J Sikdar, S Singh

**Affiliations:** Department of Orthopaedics, Maharishi Markandeshwar Institute of Medical Sciences and Research Mullana Ambala, India; Department of Orthopaedics, Maharishi Markandeshwar Institute of Medical Sciences and Research Mullana Ambala, India; Department of Orthopaedics, Maharishi Markandeshwar Institute of Medical Sciences and Research Mullana Ambala, India

## Abstract

This is a report of a study on the long term results of PHILOS plating and percutaneous K-wire fixation in a prospective series of proximal humerus fractures in elderly patients. We reviewed a total of 60 patients with proximal humerus fractures in 30 patients (Group 1), who were treated by open reduction and internal fixation with Proximal Humeral Internal Locking System (PHILOS) plate and 30 patients (Group 2) who were treated with percutaneous K-wire fixation. Functional outcome was assessed using Visual Analogue Scale(VAS) and Constant-Murley Score.

Mean Constant-Murley score was 84.6 points (range: 61- 100) in Group 1 and - 76.4 points(range:56-100) in Group 2 at final follow up. Values varied depending upon the fracture type with worst in 4-part fractures. Mean VAS Score was 2.6(range:0-10) in Group 1 and 3.8(range:0-10) in Group 2.

We obtained satisfactory results in both the groups, with each procedure having its advantages and shortcomings. We found that PHILOS plate fixation provided stable fixation with minimal implant problems and enabled early range-ofmotion exercises to achieve acceptable functional results. Fixation with percutaneous K-wires presented an efficient treatment option with the advantages of minimal invasiveness and soft tissue dissection.

**Key Words:**

Proximal humerus fractures, PHILOS plate and K-wires

## Introduction

Proximal humerus fractures are on the rise due to road traffic accidents and increase in the incidence of osteoporosis. They constitute about 4-5% of all fractures ^1^. In patients above 65 years of age these are the third most common fracture, after hip and distal radius fractures. Minimally displaced fractures, regardless of the number of fracture lines, can be treated with closed reduction but displaced fractures require anatomical reduction with internal fixation ^2,3^.

Several treatment modalities have been proposed depending upon the fracture pattern, patients’ age and level of activity, and associated medical co-morbidities: conservative treatment^4^, open reduction and internal fixation (ORIF)^5^, joint replacement^6,7^ and percutaneous fixation^7,8^. Good clinical results were seen in 92% of cases treated with ORIF, 87% for cases treated with conservative treatment and 87.5% for cases treated with shoulder arthroplasty^6,7^. Open reduction and internal fixation included extensive surgical exposure and -damage to vascular supply of bone fragments. Open reduction and internal fixation (ORIF) has the advantages of anatomical reduction and early mobilisation. It may however be associated with higher rates of infection, damage to arteries and nerves and avascular necrosis of humeral head^5^.

Conservative treatment with closed reduction and percutaneous pinning has limited indications, less blood loss, lower risk of neurovascular complications and less interferance with glenohumeral joint motion. This technique, however may not ensure anatomical reduction and early mobilisation. It is also associated with pin tract infection and a long recovery period^8^.

The aim of this study was to evaluate the long term results of PHILOS (Proximal Humerus Internal Locking System) plating and percutaneous K-wire fixation in a prospective series of proximal humerus fractures in elderly patients .

## Materials and Methods

### 

A prospective study was conducted in our institution from July 2011 to May 2013 of a total of 60 patients with proximal humerus fractures. Group 1 included 30 patients (20 males and 10 females with mean age of 65 years) who were treated with ORIF with PHILOS plate .In this group 12 patients had two-part fracture, 14 patients had three-part fracture and four patients had -four- part fracture, according to the Neer classification. Group 2 included 30 patients (16 males and 14 females with mean age of 62 years) who were treated with closed reduction and percutaneous K-wire fixation. In this group 16 patients had two-part fracture, 8 patients had threepart fracture and 6 patients had four-part fracture. Inclusion criteria for both groups were elderly patients with 2,3, or 4 part fractures. Exclusion criteria for both groups were subjects with pathological fractures, patients with primary or metastatic bone tumours, fractures with non-union and patients with neurological deficits. The mechanism of injury included road traffic accidents, fall on the ground and sportsrelated activities. Fractures of proximal humerus were classified according to Neer classification. Open fractures and those with other associated injuries were excluded from the study.

Operative technique for each group was as follows: **Group 1** Patients with proximal humerus fractures were treated with open reduction and internal fixation (ORIF) with PHILOS plate. Surgery was performed under general anaesthesia, patient in supine position with a small sand bag under the shoulder. All patients received prophylactic dose of intravenous antibiotic preoperatively. The fracture was exposed through a delto-pectoral approach and fracture fragments were reduced. The reduced fracture fragments were held in position with K-wires under guidance of image intensifier. Definitive fixation with PHILOS plate was done with the plate positioned lateral to the bicipital groove, sparing the tendon of long head of biceps. The plate was placed at least one cm distal to the upper end of greater tubercle. The required lengths of the locking screws were determined with a direct measuring device over the K-wire, and at least six locking screws were inserted in the humeral head (Figure 1a,1b).

Lesser tuberosity was fixed with separate screws or wires if found to be avulsed. Range of motion of shoulder and impingement were checked on the table. Wound was closed in layers with suction drain. Passive range of motion (ROM) exercises were initiated on the second postoperative day. Sutures were removed after 12-15 days. Active shoulder mobilization exercises were started 4 to 6 weeks postoperatively depending on the patient’s co-operation. Follow up was at one week, then every month for 6 months, and then at 12 months for final evaluation. Standard anteroposterior, axillary and lateral radiographs were obtained and evaluated for fracture healing, non-union, malunion, loosening of implant, loss of reduction and avascular necrosis of head of humerus. Clinical examination included range of motion - and strength evaluation, pain assesment according to a Visual Analogue Scale (VAS), and Constant-Murley score. The criteria for radiographic healing was when all fragments showed substantial cortical continuity.

**Group 2**Surgery was performed under general anaesthesia with the patient in beach chair position. Near anatomical reduction was achieved by manual traction and arm mobilization.Three to four threaded 2.5 mm K-wires under image intensifier were inserted depending on the number of fracture fragments. In the case of difficult reduction one K-wire of 3.5 mm (Figure 2a,2b) was used as a joystick. Care was taken on the orientation and pin placement to avoid injury to the axillary nerve, the radial nerve and the anterior circumflex humeral vessels lying medially. K-wires were left out of skin and bent at the extremity to control migration. Patients were encouraged to start active mobilisation of wrist and elbow on the second postoperative day. Dressing of the pin tracts were done on alternate days. Passive ROM exercises were initiated on the second postoperative day. Active shoulder mobilization exercises were started at 4 to 6 weeks postoperatively depending on patient’s co-operation. Follow up at one week, then every month for 6 months, and then at 12 months for final evaluation. Standard anteroposterior, axillary and lateral radiographs were obtained and evaluated for bony healing, non-union, malunion, loosening of implant, loss of reduction and avascular necrosis of head of humerus. Clinical examination included range of motion (ROM) and strength evaluation, pain assesment according to a Visual Analogue Scale (VAS), and Constant-Murley score. The criteria for radiographic healing was when all fragments showed substantial cortical continuity.

## Results

Mean operation time was 100 minutes (range 80-120 minutes) in Group 1 and 50 minutes (range 35-70 minutes) in Group 2. In Group 1 the average blood loss during surgery was 600 ml (range 400-1000 ml) whereas in Group 2 it was 100 ml (range70-160ml). Both groups received broad spectrum antibiotics postoperatively. There were no major complications intraoperatively in both the groups. One patient in Group 1 had hypotension during surgery due to excessive blood loss. This was a female patient with 3-part fracture and was managed with blood transfusions. Post operatively complications were noted in 8 patients in Group 1 and in 14 patients in Group 2 (Table 1). In Group 1 two patients had non-union (one patient with 3 part fracture and another with 4 part fracture), four patients had infection(three patients with 2 part fracture and one patient with 3 part fracture) and two had avascular necrosis of the humerus head (both these patients were with 4 part fracture). For patients with non union had bone grafting with removal of the previous implant. Patients with infection were treated with intravenous antibiotics after obtaining the culture sensitivity reports. Two patients with avascular necrosis of the head of humerus, were offered arthroplasty but they refused.

In Group 2 six patients had pin tract infection (four patients with 2 part fracture and two patients with 3 part fracture), two patients had non-union (both these patients were with 3- part fracture), four patients had malunion (three patients with 2 part fracture and one patient with 3 part fracture) and two patients had K-wire loosening (both these patients were with 2 part fracture).Patients with pin tract infection were treated with daily dressings and antibiotics, Those with non-union were treated with ORIF and bone grafting. Patients in whom the fracture had malunited did not require any treatment, as the range of movements was acceptable. The patients with K-wire loosening had removal and new wires inserted.

Mean Constant-Murley score was 84.6 points (range: 61- 100) in Group 1 and 76.4 points(range:56-100) in Group 2 at final follow up. Values varied depending upon the fracture type with the worst in 4 part fractures. Mean VAS Score was 2.6 (range:0-10) in Group 1 and 3.8 (range:0-10) in Group 2.

## Discussion

Proximal humerus fracture is the most common fracture of the shoulder. It is the second most common site of fracture in the upper limb after distal radius. These fractures have been treated with a wide range of options, namely non operative, ORIF, percutaneous screw/pin fixation and external fixation. Fractures of this region are common both with high-energy injuries in people of all ages, as well as with simple falls in older people with osteoporosis. In elderly patients fragility of the bone complicates the pattern of fracture. These patients also have comorbidities which makes the treatment of these patients even more challenging. Zyto and colleagues reported mean constant score of 65 points and no complications with conservative treatment compared with surgical approach, resulting in mean value of 60 points and with complications (avascular necrosis, infection)^4^ . Magovern, Kenner, and Nho found good constant scores with surgery and relatively few complications, with better functional scores for percutaneous fixation ^8,9,10^. Percutaneuos fixation has its limitations of poor reduction of fracture fragments, pin tract infection and long period of recovery ^8,10^. But it has the advantages of less soft tissue stripping with less exposure, less blood loss and minimal invasiveness. In cases where there is loss of reduction due to pin loosening , ORIF can be performed^10^. ORIF with PHILOS plate for treatment of proximal humerus fractures has the advantages of accurate reduction, early mobilisation, better fixation in osteoporotic bones and ease of reconstruction of comminuted irreducible fractures. On the other hand it has the disadvantages of excessive soft tissue dissection and blood loss, risks of injury to the neurovascular structures and increased risk of avascular necrosis of humeral head ^11, 12^. However, recent studies have shown good long term results of proximal humerus fractures managed by the PHILOS plate^13,14^.

In a study conducted by Fazal et al it was seen that PHILOS plate fixation provided stable fixation with minimal implant problems and enabled early range of motion exercises to achieve acceptable functional results ^15^. In the present study it was concluded that PHILOS plate provides an excellent stable construct even in multi fragmented osteoporotic proximal humerus fractures with the advantages of accurate reduction and early mobilisation. Fixation with percutaneous K-wires may -present an efficient treatment option for 2 or 3 part proximal humerus fractures with its advantages of minimal invasiveness and less soft tissue dissection. Better functional results were seen in patients treated with PHILOS plate than those treated with percutaneous K-wire fixation.

**Table I: Post-Operative Complications in Group 1 and Group 2:**
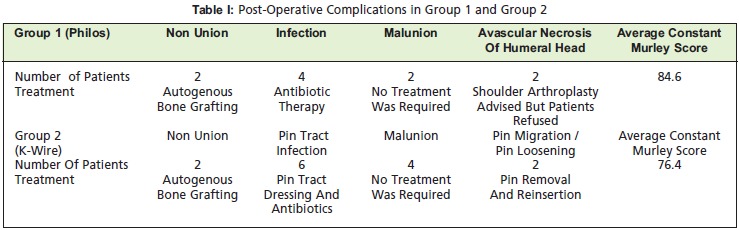


**Fig. 1a: Preoperative (A) radiograph showing 3 part fracture of proximal humerus.**
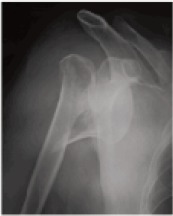


**Fig. 1b: Postoperative radiograph (B) showing fixation offracture with PHILOS plate.**
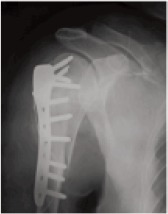


**Fig. 2a: Preoperative radiograph of proximal humerus fracture in Group 2.**
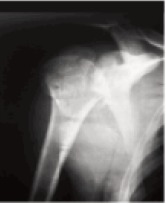


**Fig. 2b: Postoperative radiograph of fracture following K-wire fixation.**